# On Death from Chloroform

**Published:** 1873-03

**Authors:** 


### ON DEATH FROM CHLOROFORM.

(*Extract from a Recent Clinical Lecture by Dr. Erischen.*)

Thanks to the somewhat frequent occurrence of the heading, "Death from Chloroform," in the papers, the subject is beginning to excite considerable amount of interest and apprehension among the public. In the profession there has been, I think, rather a tendency to avoid the subject—to look upon the occurrence of an occasional death from chloroform as a sort of necessary price paid for the advantages of anæsthesia. Whether this be the case—whether the fatal result really depends on an inexorable fate or on some more preventable cause—is, I think, worth inquiring into. Considering the extensive and general employment of chloroform for even the most trivial of surgical operations, a death directly due to its influence is happily of rare occurrence. During the twenty-five years that I have been attached to a hospital, I have only witnessed one such death. It is a sight which must always produce a deep and painful impression on those present; the more so, since the administration of chloroform is by no means a necessary part of the operation. The relief of pain is in many cases nothing more than a luxury.

All surgeons will agree with me that in extra hospital practice, especially, the administration of chloroform is that part of the operation which often gives most anxiety to the operator. In a hospital, chloroform is generally administered by some one who is in the daily habit of performing that duty. The process is watched by competent observers, and there is every appliance at hand in case of need. In private practice it is often given by the practitioner in charge, whose only experience is derived from a limited use of the drug in midwifery cases, and both the patience and peace of mind of the surgeon is upset; for indeed a considerable amount of practice and experience are required to enable a man to chloroform well. Some acquire the necessary skill more easily than others, but no amount of care can make up for the want of a certain amount of practice.

We must confine our attention to deaths which are *directly and immediately* due to chloroform. These may occur in three ways—by the lungs, by the head, or by the heart; from asphyxia, from coma, or from syncope.

1. *Asphyxia* may be caused, first, by tight clothing, or by any thing which hinders the respiratory movements, as a large abdominal tumor; second, by actual choking. If chloroform be given too soon after a meal, vomiting of semi-digested material is very likely to occur, and the larynx may be obstructed by it. A gag or false teeth may slip into the throat and cause choking. The tongue also, like the other voluntary muscles of the body, becomes paralyzed when the patient is fully anæsthetized, and by falling back into the throat, may itself cause choking. Deaths from these causes, the first more especially, have occurred most often in dentists' practice. Simple asphyxia is not often actually fatal, though narrow escapes are common. The signs are so well marked that they can scarcely be overlooked, and the patient can generally be recovered if assistance be at hand.

2. Death from *coma* due to chloroform is rare; still the only case with which I have been personally connected was due to this cause. The patient was suffering from chronic Bright's disease, and had slight symptoms of uræmia. The administration of chloroform had not proceeded far when convulsions occurred, followed by coma and death in about an hour. In this case, at all events, the mode of death was evidently predisposed to by the poisoned state of the blood.

3. We come at last to that part of the subject to which I wish more particularly to direct your attention—the death from *cardiac syncope*, as it is called. This is to me a very puzzling mode of death, as difficult to account for as it is to guard against. I presume that by the term cardiac syncope is meant atony and failure of the heart's action. This is an easy explanation, but not altogether a satisfactory one. Among these numerous experiments which have been made with chloroform, I do not know of any which prove that it has any direct syncopal action on the heart, or even any indirect toxic action on that organ through its nerves. Still there is no doubt of the fact that people do die without much disturbance of respiration—without becoming distinctly livid in the face. The pulse fails—that is the first thing noted—and then they are dead.

Now my own idea is these are really cases of asphyxia; that the heart is secondarily, not primarily, affected; and my explanation would be as follows: after death, we find in these cases a weak, fatty heart; the valves indeed healthy, but the walls thin, and the muscular tissue pale and degenerated. Now chloroform has always, especially at first, a slight asphyxial tendency. The patient calls out that he is choking, tries to pull away the inhaler, and breathes deeply, then struggles and holds his breath for a few seconds. In a healthy man this soon passes off—the inconvenience is merely temporary; but with a fatty, enfeebled heart, it is different. The patient holds his breath for a few seconds, the right side of the heart is soon filled, there is weak propulsive power, the organ can not recover itself, and the result is fatal.

Some years ago I made numerous experiments on death by asphyxia in animals; and I found that if once the ventricular action were stopped—if a contraction were missed—it was most difficult to start again; generally all was over. And this is what happens in these cases: the ventricle can not be emptied quickly enough, the rythm is lost, and almost instantaneous death ensues. The patient then does not die from the direct action of the chloroform on the heart, but from the effect of a slight asphyxial condition, which is inseparable from the administration of this agent upon a disorganized heart. After death you may not find the right side of the heart greatly engorged; first, because great engorgement is not necessary to cause a fatal result; and secondly, the blood, even many hours after death from chloroform, is found unusually fluid, so that the dependent parts of the body are congested, and the heart is left comparatively empty. Then also artificial respiration is always set up in these cases, and this tends to diminish the cardiac plethora.

Finally, a few cautions: Never give chloroform without first thoroughly loosening the dress; if possible, not within four hours of a meal; the head should be moderately raised; the pulse, respiration and color of the face must be carefully watched. As regards the occurrence of the rigid spasm already adverted to, I do not think that sufficient attention has been directed to this condition. A patient with an enfeebled heart is then in a most dangerous, even critical, state; the chest walls are fixed, the lungs are filled with chloroform vapor, which become diffused, but can not escape; the pulmonary circulation is obstructed, and the pressure on the right ventricle rapidly increased. Let the patient really recover, then give it again slowly; watch for any blueness around the mouth, etc. If the pulse fail, draw forward the tongue with forceps, and set up artificial respiration at once; but prevention is the main point. When there is well-marked asphyxia, artificial respiration is most useful and generally successful; but in these cases of so-called "cardiac syncope" it is generally useless. The heart has been barely able to meet the ordinary requirements of the system; it can not recover lost ground; it gives in, as it were, at once.—*American Practitioner.*

